# Levodopa–carbidopa intrajejunal infusion in Parkinson’s disease: untangling the role of age

**DOI:** 10.1007/s00415-020-10356-x

**Published:** 2020-12-22

**Authors:** Francesca Morgante, Valentina Oppo, Margherita Fabbri, Enrica Olivola, Chiara Sorbera, Rosa De Micco, Giovanna Chiara Ielo, Fabiana Colucci, Salvatore Bonvegna, Alessio Novelli, Nicola Modugno, Mariachiara Sensi, Maurizio Zibetti, Leonardo Lopiano, Alessandro Tessitore, Manuela Pilleri, Roberto Cilia, Antonio E. Elia, Roberto Eleopra, Lucia Ricciardi, Giovanni Cossu

**Affiliations:** 1 Neurosciences Research Centre, Molecular and Clinical Sciences Research Institute, St George’s University of London, Cranmer Terrace, London, SW17 0RE United Kingdom; 2 Department of Experimental and Clinical Medicine, University of Messina, Messina, Italy; 3 Movement Disorders and Neurophysiology Unit, Department of Neuroscience, AO Brotzu, Piazzale Ricchi 1, Cagliari, 09134 Italy; 4 Department of Neurosciences, Clinical Investigation Center CIC 1436, Parkinson Toulouse expert center, NS-Park/FCRIN network and NeuroToul COEN center, TOULOUSE University Hospital, INSERM, University of Toulouse 3, Toulouse, France; 5 Unit of Neurology, IRCCS Neuromed, Pozzilli, IS Italy; 6 Neurorehabilitation Unit, IRCCS Centro Neurolesi “Bonino Pulejo,”, Messina, Italy; 7 Department of Advanced Medical and Surgical Sciences, University of Campania “Luigi Vanvitelli”, Naples, Italy; 8 Service of Neurology, Private Hospital, Villa Margherita-Santo Stefano, Arcugnano, Italy; 9 Department of Neuroscience and Rehabilitation, Azienda Ospedaliera-Universitaria S. Anna, Ferrara, Italy; 10 Movement Disorder Unit, Fondazione IRCCS Istituto Neurologico Carlo Besta, Milan, Italy; 11 Department of Neuroscience “Rita Levi Montalcini”, University of Torino, Turin, Italy; 12 MRC Brain Network Dynamics Unit, Nuffield Department of Clinical Neurosciences, Oxford, UK

**Keywords:** Parkinson’s disease, Motor fluctuations, Dyskinesia, Levodopa–carbidopa intestinal gel (LCIG), Old age, Quality of life

## Abstract

**Objectives:**

Levodopa–Carbidopa Intrajejunal gel (LCIG) infusion is an effective intervention for people with advanced Parkinson’s disease (PD). Although age may not be a limiting factor for LCIG implant, no data are available on late elderly PD (LE-PD) subjects. In this cross-sectional, we aimed to demonstrate if older age may impact on quality of life (QoL), motor and non-motor symptoms severity, and profile of side effects in PD treated with LCIG.

**Methods:**

Out of 512 PD subjects treated with LCIG at 9 Italian PD centers, we selected 25 LE-PD defined as age ≥ 80 years at last follow-up who were available to attend the study visit. Twenty-five PD patients (Control-PD, defined as age < 75 years at last follow-up) matched to LE-PD by disease and LCIG duration served as control group. The following motor and non-motor variables were ascertained: quality of life (PDQ-8), time spent in ON, wearing-off Questionnaire, Unified PD Rating Scale, freezing of gait questionnaire, Parkinson’s disease sleep scale-2, Non Motor Symptoms Scale (NMSS), and MOCA.

**Results:**

No statistically significant differences were found between LE-PD and Control-PD on PDQ-8 and several motor and non-motor variables. LE-PD had less frequent and milder impulsive–compulsive behaviors and milder dyskinesia. At multivariable regression, worse quality of life was associated with UPDRS-III and NMSS scores but not to age at study visit and age at LICG implant. Rate of adverse effects was similar in both groups. Drop-out rate calculated in the whole PD cohort was comparable between the two groups.

**Conclusion:**

Our data provide evidence that valuable LCIG infusion might be achieved in late elderly PD.

**Supplementary Information:**

The online version contains supplementary material available at 10.1007/s00415-020-10356-x.

## Introduction

Advanced Parkinson’s disease (PD) encompasses a wide spectrum of motor and non-motor symptoms which ultimately affect the choice of treatment aimed to improve quality of life. The presence of disabling motor fluctuations and/or dyskinesia, despite adjustment of oral medication, represents an indication for device-aided treatments, such as Deep Brain Stimulation (DBS), continuous subcutaneous apomorphine (CSAI), and levodopa–carbidopa intestinal gel (LCIG) infusion. Accordingly, best outcomes occur in younger PD subjects in whom axial symptoms, such as gait and balance disturbances, are not prominent [14, 24]. Given the increased incidence of PD in the elderly population and prolonged survival as a consequence of dopaminergic replacement treatment [16], it is expected that a proportion of patients with long disease duration, motor complications, and relatively preserved cognitive features might be suitable for device-aided therapies also in very old age. Older population is growing in industrialized countries and outcomes of different pharmacological interventions from cohorts of “late elderly” (LE) subjects are increasingly reported, at least for dementia [10].

Older age is a limiting factor for DBS eligibility [2, 15], as in the elderly population the effect on quality of life is not significant [6, 26] and age is associated with higher risk of post-operative complications [21]. No data have been reported so far about safety of CSAI and LCIG in elderly cohorts (i.e., ≥ 80 years) and their impact on quality of life, despite a recent Delphi consensus of movement disorders experts have suggested that age may not be a limiting factor [2]. A post hoc analysis of the GLORIA registry reported improvement of quality of life (QoL) in younger and older patients under LCIG, but cut-off was set at age 65 and evaluation of non-motor symptoms was not reported [1]. One retrospective study conducted in a CSAI cohort showed that efficacy, rated by clinical global impression, was comparable in younger and older PD patients [17]. Yet, a few studies suggested that older age might be a factor leading to discontinuation of infusion therapies [3, 17, 19, 25]. Accordingly, a higher risk of dropout from LCIG infusion, especially in the first year of treatment, was found in patients older than 70 years due to surgery, device, and infusion-related complications [3]. For CSAI, factors leading to discontinuation in older patients were side effects, mainly neuropsychiatric [25], worsening of cognition, and lack of control of dyskinesia [17].

In the present study, we tested the hypothesis whether older age may impact on QoL, motor and non-motor symptoms severity, and profile of side effects in PD subjects treated with LCIG. We predicted that late elderly Parkinson’s disease patients implanted with LCIG because of disabling motor complications will have similar QoL to younger PD subjects. To this aim, we designed a cross-sectional study comparing LE-PD patients (age ≥ 80 years) to a control PD group (age < 75 years) matched by disease duration and time receiving LCIG.

## Methods

This was a cross-sectional study with a retrospective analysis of adverse effects and quality-of-life pre-LCIG. PD patients who underwent LCIG infusion for at least 6 months were enrolled from nine Italian tertiary referral centers for PD. Clinical evaluations were performed at the time of study visit which corresponded to the latest follow-up after LCIG.

Inclusion criteria for LCIG at the time of implant were: a diagnosis of PD according to MDS diagnostic criteria [18]; presence of severe motor fluctuations and/or dyskinesia despite best medical treatment; absence of severe psychosis or severe hallucinations; absence of systemic diseases which may interfere with the device and LCIG therapy; absence of dementia (by DSM-IV criteria). We defined subjects aged ≥ 80 years as “late elderly PD” (LE-PD) and subjects aged < 75 years as “Control-PD”. This cut-off was chosen based on previous studies in elderly cohorts [10] which defined 75 years as the cut-off to distinguish middle elderly (< 75 years) from late elderly (> 75 years). We raised the threshold for late elderly to ≥ 80 years to further distinguish the two cohorts. From the whole LCIG cohort actively followed up in each center), we selected LE-PD patients available to attend study visits. Subsequently, we identified Control-PD patients matched by disease duration and LCIG duration.

Institutional ethics approval was obtained in the Coordinator Centre (AOU Brotzu, Cagliari, Pr. PG 2017/17817) and approved by the ethical Committees of each participating centers. Each participant provided written informed consent before study participation.

### Demographical and clinical variables

For each subject, the following demographic and clinical data were collected at the time of study entry: age, gender, education level, weight, age at disease onset, disease duration, most affected side, LCIG duration, and all type and number of current comorbidities (at the time of study inclusion). Charlson Comorbidity Index (CCI) was also employed to categorize comorbidities.

Quality of life was assessed by means of the Parkinson’s Disease Questionnaire-8 (PDQ-8) summary index. For motor symptoms, we employed: Unified Parkinson’s Disease Rating Scale parts II, III (in the ON state) and IV, respectively, for disease severity and complication of therapy; Rush Dyskinesia rating scale; Hoehn–Yahr stage; freezing of gait questionnaire (FOG-Q); time spent in ON (hours/day, based on therapy’s diary). PDQ-8 and time spent in ON (hours/day) were also retrospectively retrieved at the time before having LCGI (as per LCGI protocol in the centers participating in this study).

Motor and non-motor fluctuations were screened by means of the 19-items Wearing Off Questionnaire (WOQ-19). Non-motor symptoms were assessed with Non-Motor Symptoms Scale (NMSS). Parkinson’s Disease Sleep scale-II (PDSS-II) and Epworth Sleepiness scale (EDS) were, respectively, employed to rate sleep and excessive daytime sleepiness. Overall cognitive function was tested by means of the Montreal Cognitive Assessment (MOCA). For impulsive–compulsive behavior (ICB) diagnosis, we employed a semi-structured interview based on diagnostic criteria for pathologic gambling, compulsive buying, compulsive sexual behavior, binge eating, punding, and compulsive use of dopaminergic therapy. In addition, the Questionnaire for Impulsive–Compulsive Disorders in Parkinson’s Disease–Rating Scale (QUIP-RS) was employed. Total and impulse control disorder (ICD) score were calculated [28]. Questionnaires from patients with dementia were filled by their caregivers.

Information about PD medications at the time of study visit was collected and levodopa equivalent daily dose (LEDD) and dopamine-agonist equivalent dose (D-Ag LEDD) were calculated [27]. LEDD and D-Ag LEDD before LCIG were also retrospectively retrieved from medical records review. Infusion parameters were also recorded, including morning (ml) and infusion (ml/hour) dose, bolus dose (ml), and number of bolus doses per day.

### Adverse effects

We collected surgery-related, device-related, and LCGI related adverse effects, since implant and up to latest follow-up at study visit. Surgery and device-related complications included stoma infections and granulomatosis, abdominal pain, incision site erythema, complications of device insertion, post-operative delirium, peritonitis, duodenal ulcer, and failure of the infusion system. Tube-related adverse effects included dislocation, coiling, kinking, and occlusion of the intestinal tube. Therapy-related side effects included hallucinations, polyneuropathy (graded as mild, moderate, severe based on nerve conduction studies and electromyography), delirium, weight loss, taste impairment, and presence of moderate/severe dyskinesia. Dyskinesia were defined as moderate/severe if item 33 of UPDRS-IV was ≥ 2. For each of the adverse effects, the number of events over all follow-up was calculated.

### Drop-out rate calculation in the whole LCIG cohort

Analysis of the drop-out rate was performed in the whole LCIG population followed-up at each participating center to verify whether LE-PD subjects included in the studies might be representative of a general LE-PD population treated with LCIG.

## Statistical methods

After checking for normal distribution of the variables by Kolmogorov–Smirnov test, groups’ comparisons were performed by either *t* test or Mann–Whitney for continuous variables and Chi-square or Fisher’s exact test for categorical data. For total and D-Ag LEDD, PDQ-8, time in ON analysis, we also performed repeated-measure ANOVA (R-ANOVA) with *“time”* (two levels: pre-LCGI, post-LCGI) as within-subjects factor and *“group”* (two levels: LE-PD, Control-PD) as between-subjects factor. Conditional on significant *F* values, we performed post hoc pair-wise comparisons by *t* test in each group.

QUIP-RS and PDQ-8 at last follow-up after LCIG were employed in univariable linear logistic regression analysis to explore their relationship with demographic and clinical variables. Parameters that were significantly associated with the outcome in the univariable analyses were then included in a multivariable model. Univariable binary logistic regression analysis was also employed to explore the association between presence of ICB and demographic and clinical variables.

Significance level was set at *p* < 0.05. Statistical analysis was performed with SPSS Statistics, version 25. Data are shown as mean and standard deviation (SD).

## Results

Out of 512 PD subjects implanted with LCIG, we enrolled 25 patients LE-PD to whom 25 Control-PD patients were matched. As per study inclusion criteria, the two groups were matched by disease duration and LCIG duration and differ by age at study entry (Table 1). The number of patients with comorbidities was higher in LE-PD compared to Control-PD. Mean duration of LCIG treatment was approximately 5 years in both groups. Daily LCIG dose and morning LCIG dose did not differ between groups. No differences were found in education level, whereas there was a trend for MOCA to be lower in LE-PD. At the time of evaluation, 5 subjects satisfied DSM-IV criteria for dementia (*n* = 3 LE-PD, *n* = 2 Control-PD).

**Table 1:** Demographical and Clinical data in Late Elderly (LE) and Control Parkinson’s Disease subjects treated with Levodopa–Carbidopa Intrajejunal gel (LCIG) infusion

	LE-PD mean ± SD (range)	Control-PD mean ± SD (range)	*p *value
Age (years)	81.48 ± 1.73 (80–85)	69.20 ± 5.45 (51–74)	< 0.0001*
Education (years)	8.76 ± 4.05 (3–18)	8.8 ± 4.57 (5–18)	0.945
Disease duration (years)	21.28 ± 7.93 (10–51)	21.28 ± 5.97 (13–38)	0.409
Weight (kg)	61.56 ± 13.41 (33–95)	65.48 ± 17.24 (40–120)	0.409
LCIG duration (months)	59.28 ± 41.62 (4–133)	60.08 ± 37.42 (6–146)	0.846
LCIG dose (ml/day)	45.098 ± 13.15 (21–73.2)	52.336 ± 18.96 (20.4–82.1)	0.148
Morning dose (ml)	4.608 ± 2.72 (0–10.5)	5.188 ± 2.69 (0–10.5)	0.341
UPDRS-II	24.6 ± 9.18 (9–42)	20.36 ± 8.91 (5–39)	0.112
UPDRS-III	39.833 ± 13.61 (21–74)	33.08 ± 14.28 (11–65)	0.107
UPDRS-IV	6.8 ± 3.74 (1–14)	8.92 ± 6.01 (4–34)	0.157
HY	3.74 ± 0.98 (2–5)	3.18 ± 0.97 (1.5–5)	0.062
RDRS	6.04 ± 4.07 (0–12)	9 ± 5.21 (0–18)	0.047*
WOQ-19 total	7.44 ± 5.01 (0–17)	7.44 ± 4.22 (0–20)	0.977
WOQ-19 motor	4.92 ± 3.05 (0–11)	5.08 ± 2.41 (0–10)	0.961
WOQ-19 non-motor	2.88 ± 3.55 (0–15)	2.36 ± 2.50 (0–10)	0.861
NMSS	78.72 ± 46.47 (19–201)	81.64 ± 45.16 (17–188)	0.749
QUIP-RS^a^	7.50 ± 3.62 (3–12)	15.07 ± 10.08 (5–40)	0.05*
FOG-Q	14.083 ± 6.83 (3–26)	13.84 ± 6.01 (3–24)	0.936
PDSS-2	9.924 ± 1.98 (4–39)	18 ± 11.73 (2–49)	0.594
EDS	5.8 ± 5.33 (0–20)	6.48 ± 5.3 (0–19)	0.449
MOCA	17.52 ± 7.2 (2–28)	20.84 ± 6.58 (2–29)	0.060

*FOG-Q* freezing of gait questionnaire, *EDS *Epworth Sleepiness scale, *HY *Hoehn–Yahr stage, *L *Levodopa–Carbidopa intestinal gel infusion, *MOCA *Montreal Cognitive Assessment, *NMSS *Non-Motor Symptoms Scale, *PDSS-2 *Parkinson’s Disease Sleep scale-II, *QUIP-RS *Questionnaire for Impulsive–Compulsive Disorders in Parkinson’s Disease–Rating Scale, *RDRS *Rush Dyskinesia rating scale, *UPDRS *Unified Parkinson’s Disease Rating Scale, *WOQ-19 *Wearing Off Questionnaire 19 items

^a^QUIP-RS calculated on LE-PD (*n* = 6) and Control-PD (*n* = 14) with impulsive–compulsive behavior

*Significant values are bolded

**Fig. 1:**
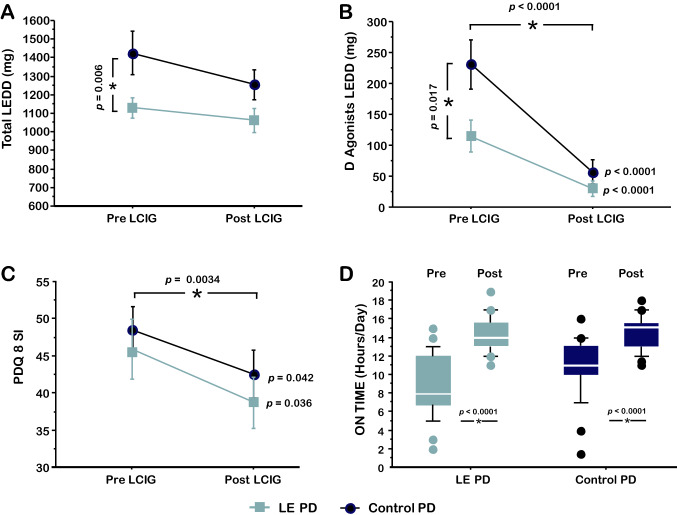
Dopaminergic medications, quality of life, and time in “on” in LE-PD and Control-PD. Total and Dopamine-agonists (D-Ag) Levodopa Equivalent Daily Dose (LEDD) (**a**, **b**), PDQ-8 summary index (SI) and time spent in ON (**c**, **d**) before and after Levodopa–Carbidopa Intrajejunal Gel Infusion (LCIG) in “Late elderly” Parkinson’s disease (LE-PD) and Control-PD. After LCIG, a significant decrease of D-Ag LEDD and PDQ-8 and a significant increase of time spent in ON were achieved, without differences between LE-PD and Control-PD

Total LEDD and D-Ag LEDD before LCIG were significantly lower in LE-PD. LCIG was associated with a significant improvement of quality of life, prolongation of time spent in ON, and a similar decrease of D-Ag after LCIG in both groups compared to baseline (Fig. 1).

With respect to motor and non-motor variables, we did not find any significant difference between LE-PD and Control-PD, except for dyskinesias which were more pronounced in Control-PD (*p* = 0.0477) (Table 1).

**Fig. 2:**
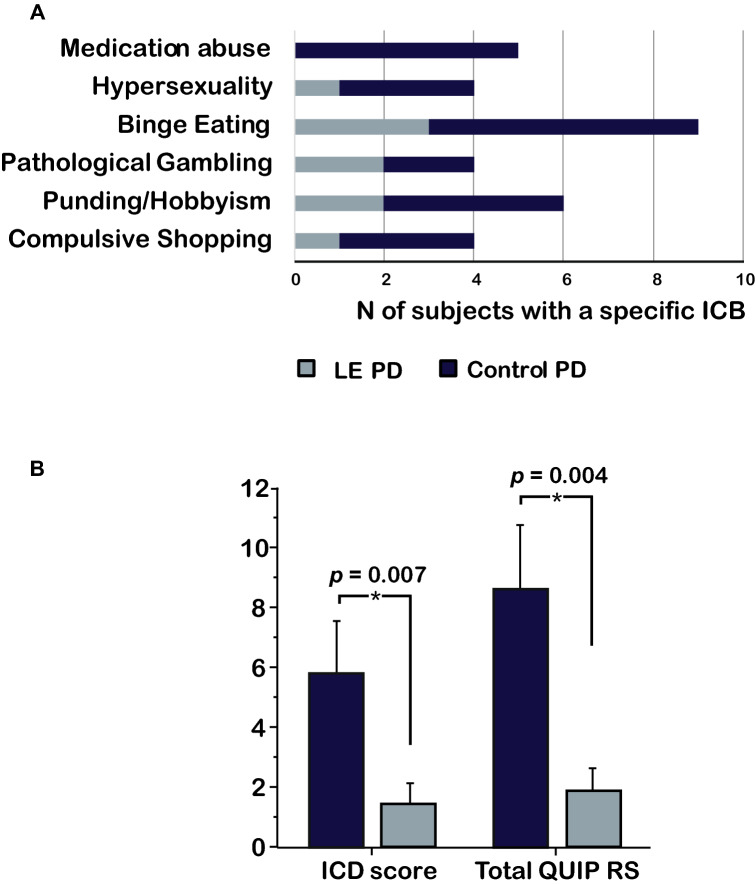
Impulsive compulsive behaviors in LCIG: effect of age. Distribution of different types of impulsive–compulsive behaviors (ICB) in “Late elderly” Parkinson’s disease (LE-PD) and Control-PD (**a**). QUIP-RS total score and ICD score were significantly higher in LE-PD compared to Control-PD (**b**). *Mann–Whitney *U* test

ICB was more frequent in Control-PD (*N* = 14) than in LE-PD (*N* = 6) (*p* = 0.04). Mean number of ICB per patient was comparable (LE-PD = 1.5 ± 0.54; Control-PD = 1.71 ± 0.91). Figure 2a shows distribution of specific ICBs in each group. Binge eating (*N* = 6) and dopaminergic medication abuse were the most frequent ICB in Control-PD (*N* = 5). Total QUIP-RS score and ICD score of QUIP-RS (calculated in subjects diagnosed with ICB) were higher in Control-PD than LE-PD (Fig. 2b).

### Regression analysis

PDQ8 after LCIG was associated with UPDRS-II, UPDRS-III, and NMSS but not with age at study visit and age at implant by univariable linear regression analysis (supplementary Table 1). At the multivariable linear analysis, only UPDRS-III and NMSS significantly predicted PDQ-8 (Table 2). Using the enter method UPDRS-II, UPDRS-III and NMSS explained significant amount of the variance in the value of PDQ-8 (*F*(3,45) = 7.19, *p* =  < 0.0001, R2 = 0.32, R2 Adjusted = 0.28).

**Table 2:** Multivariable regression analyses with PDQ8, QUIP-RS, and ICB diagnosis as dependent variables

	B	95% CI–LB	95% CI–UB	p Value
Multivariable regression with PDQ8 after LCIG as dependent variable				
UPDRS-II	− 0.474	− 1.185	0.236	0.185
UPDRS-III	**0.581**	**0.124**	**1.038**	0.014*
NMSS	**0.135**	**0.035**	**0.235**	0.009*
Multivariable binary regression with ICB diagnosis as dependent variable				
Group	**1.747**	0.037	0.813	0.026*
D-Ag LEDD	0.007	0.996	1.017	0.215
HY	− 0.09	0.391	2.135	0.836
NMSS	− **0.027**	0.951	0.996	0.022*
Multivariable linear regression with QUIP-RS as dependent variable				
Age at onset	− 0.138	− 0.444	0.169	0.37
Group	− **8.533**	− **16.703**	− **0.364**	0.041*
Age	0.362	− 0.275	0.999	0.258
HY	− 2.191	− 4.648	0.266	0.079

*CI *confidence interval, *D-Ag *dopamine agonists, *HY *Hoehn–Yahr stage, *ICB *impulsive–compulsive behaviors, *LB *lower bound, *LCIG *Levodopa–Carbidopa intestinal gel infusion, *LEDD *levodopa equivalent daily dose, *NMSS *Non-Motor Symptoms Scale, *UB* = upper bound, *UPDRS *Unified Parkinson’s Disease Rating Scale

*Significant values are bolded

Univariable linear regression analysis with Rush Dyskinesia Rating Scale as dependent variable showed that only age at onset was a significant regressor (*B* = − 0.154; 95% CI (− 0.293, − 0.015); *p* = 0.03).

Univariable binary logistic regression showed that diagnosis of ICB was associated with Control-PD group (*p* = 0.024), D-Ag LEDD post-LCIG (*p* = 0.004), and it was negatively associated with Hoehn–Yahr stage (*p* = 0.023) and NMSS (*p* = 0.01). At multivariable level, Control-PD group (*p* = 0.026) and NMSS (*p* = 0.022) were the only significant regressors for a diagnosis of ICB (Table 2). The four independent variables (Group, D-Ag LEDD, HY, and NMSS) in the logistic model together account for 31% the explanation for the diagnosis of ICB (Cox & Snell R Square = 0.31). Univariable linear regression showed that QUIP-RS score was inversely associated with age, age at onset, Hoehn–Yahr stage, and Control-PD group (Supplementary Table 2). However, at the multivariable analysis, only group predicted the severity of QUIP-RS, confirming that Control-PD was associated with more severe ICB. Age at onset, group, age, and H&Y explained significant amount of the variance in the value of QUIP-RS (*F*(4,45) = 3.3, *p* = 0.01, R2 = 0.22, R2Adjusted = 0.16).

### Adverse effects

Frequency of adverse effects was comparable between the two groups (Table 3). In general, patients experienced less than 5 adverse events from implant to last follow-up (Supplementary Fig. 1). Incision site erythema or granulomatosis, tube dislocation, weight loss, hallucinations, and moderate/severe dyskinesia were the adverse effects experienced more commonly. Life-time frequency of surgery and device-related complications were comparable among LE-PD (16/25) and Control-PD (19/25) (*p* = 0.54). Frequency of adverse effects did not differ across centers (*p* < 0.05).

**Table 3:** Adverse Reactions (ADR) in Late Elderly (LE) and Control Parkinson’s Disease subjects treated with Levodopa–Carbidopa Intrajejunal gel infusion

	LE-PD (Y/N)	Control-PD (Y/N)	*p *value
ADR (> 5 events per patient)	5/20	8/17	0.5
Stoma infection	3/22	7/18	0.289
Abdominal pain	0/25	0/25	> 0.9999
Incision site erythema or granulomatosis	10/15	12/13	0.7761
Complications of device insertion	1/24	2/23	> 0.9999
Peritonitis	0/25	0/25	> 0.9999
Duodenal ulcer	0/25	1/24	> 0.9999
Failure of the infusion system	2/23	5/20	0.4174
Tube dislocation	10/15	6/19	0.3635
Tube coiling, kinking and occlusion	9/16	10/15	> 0.9999
Unintentional removal of tube by the patient	1/24	6/19	0.0983
Hallucinations	7/18	6/19	> 0.9999
Delusions	3/22	3/22	> 0.9999
Polyneuropathy	8/17	7/18	> 0.9999
Delirium	3/22	3/22	> 0.9999
Weight loss	12/13	12/13	> 0.9999
Taste impairment	0/25	2/23	0.4898
Moderate/severe dyskinesia	9/16	11/14	0.7733

### Drop-out rate in the whole LCIG cohort

We retrospectively compared the number of subjects who dropped out from LCIG in Control-PD and LE-PD. Out of 506 PD patients with available clinical data until last follow-up (*n* = 6 were lost to follow-up), there were 111 drop-outs (21.75%) and 80 deaths (15.6%) (LE-PD = 47/191, 24.6%, Control-PD = 33/315, 10.4%, *p* = 0.00002). Drop-outs were more frequent in Control-PD (82/315, 26.0%) compared to LE-PD (29/191, 15.1%, (*p* = 0.038). There was a significant difference in the rate of drop-outs across participating centers (*p* < 0.0001; range of drop-out rate: 6–50%). Duration of LCIG treatment at drop-out was comparable in LE-PD (1.8 ± 1.5 years) and Control-PD (2.8 ± 2.2 years) (*p* = 0.07). Polyneuropathy and lack of compliance with LCIG management were the most frequent causes of drop-out in Control-PD, whereas in LE-PD device-related adverse events, lack of benefit and dementia were the most frequent causes (Supplementary Fig. 2).

## Discussion

Despite strong evidence of efficacy of LCIG and no age limit recommendation for this treatment [2], studies reporting data on subjects older than 80 are lacking. Older patients with advanced PD are often not informed on the available advanced therapies, including LCIG, or are denied the screening for advanced therapies because of older age [12]. PD without dementia calculated on January 1, 2006 in Olmsted County had a prevalence of 1.39% in the age group 80–89 years, and constituted 56.4% and 47.6% of subjects with PD in that age limit, respectively, in men and women [22]. Based on these data, it could be estimated that an increasing number of people with PD aged > 80 will be in an advanced stage of the disease without dementia and might benefit from device-aided therapy.

Our data provide evidence that both age at last follow-up and age at implant do not impact on quality of life and the severity of a wide spectrum of motor and non-motor symptoms in PD treated with LCIG at a mean follow-up time of 5 years. Indeed, UPDRS-III and NMSS were the only factors associated to worst quality of life under LCIG treatment. More importantly, profile of side effects of LCIG infusion was comparable across age groups.

These results should be evaluated, considering that our LE-PD cohort is a specific population of older PD subjects who satisfied selection criteria for LCIG and in whom the number of accumulated deficits expected for that age is lower and contribute to less frailty and success of such therapy [11]. In our study, both LE-PD and Control-PD had mean disease duration of 21 years. This implies that in LE-PD, the disease has started in the early old age and progressed to develop disabling motor complications in a later old age. Whereas response to oral Levodopa has been assessed for PD patients in late stage [8], studies assessing LE-PD with long disease duration who still have prominent response to Levodopa with fluctuations and dyskinesia are lacking. This is a fundamental issue to clarify, as this population could still benefit from infusion therapies, if patients met the inclusion criteria. In younger patients with disease onset before age 50 and a disease duration of up to 30 years, despite worsening of axial and cognitive symptoms, response to oral levodopa and STN DBS has been proved [13]. Here, we showed a similar pattern for those subjects whose disease onset occurred at younger age or earlier elderly age and who slowly progressed to have severe motor fluctuations in late elderly age. Despite similar scores of PDQ-8 and duration of ON time before LCIG, LE-PD were treated with significant less dopaminergic medications before LCIG. This might reflect the vulnerability of this age group for psychiatric side effects and the use of lower doses of dopaminergic medication to prevent them [5]. At this regard, the decrease of dopamine agonists made possible by LCIG is an opportunity for this age group to improve their motor status without producing adverse psychiatric side effects.

One of the major issues with LCIG therapy is the relatively high frequency of device-related and infusion-related complications, likely determining more frequent drop-outs in patients older than 70 [3]. Our data showed a similar rate for surgery, device, and infusion-related side effects in LE-PD and Control-PD over a long follow-up time. The majority of the events were mild and only 20% and 32% of subjects with, respectively, LE-PD and Control-PD experienced more than 5 events since the implant of LCIG. Nevertheless, the high rate of tube dislocation, coiling, kinking, and occlusion in our study cohort support the view that these side effects are often manageable, especially when a multidisciplinary team is involved [23], and do not lead to discontinuation. Also, we reported less frequent drop-outs in LE-PD, with number of deaths being higher in this group, as expected. These data further support the feasibility and management of LCIG in late elderly.

Another novel data of our study are that under LCIG treatment, LE-PD had less frequent and severe ICB and less severe dyskinesia, despite comparable frequency of patients perceived disabling dyskinesia. The only predictor of dyskinesia severity was age at onset, confirming the relevance of this variable on the expression of levodopa-induced dyskinesia [7].

In a 6-month prospective observational study, the change in ICB severity after LCIG was independent of dopamine-agonist treatment [4]. In our sample, whereas, at the univariable analysis, dopamine-agonist dose was associated with the diagnosis of ICB, at the multivariable level, the only predictors of ICB diagnosis were an age < 75 and severity of non-motor symptoms. The association of ICB with younger age is well known, whereas, only recently, it has been demonstrated that PD patients with ICB are more likely to present specific non-motor symptoms, such as dysautonomia [20] or REM behavioral sleep disorder [9].

The main limitations of the present study are small sample size and the lack of longitudinal assessment of motor and non-motor variables. Nevertheless, the comparability of quality of life at last follow-up and the lack of association between age and quality of life at univariable and multivariable analyses support the interpretation that, under LCIG treatment, age does not impact on quality of life and motor and non-motor severity. Moreover, the strict matching for disease duration and LCIG duration of our study cohort is a strength, which has minimized the effect of these important confounders. Finally, the long follow-up time of our study cohort enabled us to have a broad view of the profile of adverse events.

In conclusion, our data suggest that advanced PD patients that fulfill the general inclusion criteria for LCIG therapy [2] are suitable candidates for LCIG treatment independently of their age. Prospective studies on LCIG in elderly PD cohorts are required to further strengthen this hypothesis and allow late elderly PD to receive a treatment which improves their quality of life.

## Data access and responsibility statement

F. Morgante had full access to all the data in the study and takes responsibility for the integrity of the data and the accuracy of the data analysis.

## Supplementary Information

Below is the link to the electronic supplementary material.

Supplementary file1 (DOCX 47 KB)
