# Supplementary material for: Levodopa–carbidopa intrajejunal infusion in Parkinson’s disease: untangling the role of age
Source: J Neurol. 2020 Dec 22;268(5):1728–37. doi: 10.1007/s00415-020-10356-x (PMC8068706; doi:10.1007/s00415-020-10356-x)
Supplement: Supplementary file 1 — Supplementary file1 (DOCX 47 KB) [file 415_2020_10356_MOESM1_ESM.docx]

**Morgante et al, LEVODOPA CARBIDOPA INTRAJEJUNAL INFUSION IN LATE ELDERLY PARKINSON’S DISEASE: A CASE CONTROL STUDY**

**Supplementary Material:**

**- Supplementary Figure 1**

**- Supplementary Figure 2**

**- Supplementary Table 1**

**- Supplementary Table 2**

**Supplementary Figure 1.** **SIDE EFFECTS FROM LCIG AND EFFECT OF AGE.**

Distribution of number of side effects in each subject with late elderly Parkinson’s disease (LE-PD) and Control-PD.

**Supplementary Figure 2. DROP-OUTS FROM LCIG: EFFECT OF AGE.**

**Distribution of drop-outs in LE-PD and Control-PD and associated causes**

**Supplementary table 1: Univariable regression analysis with PDQ8 after Levodopa Carbidopa Intrajejunal gel infusion (LCIG) as dependent variable**

|  | B | 95.0% CI for B | | P-Value |
| --- | --- | --- | --- | --- |
|  |  | **Lower Bound** | **Upper Bound** |  |
| Age | -0.024 | -0.672 | 0.623 | 0.9 |
| Age at implant | -0.044 | -0.636 | 0.547 | 0.5 |
| Disease duration | 0.298 | -0.388 | 0.983 | 0.4 |
| LCIG duration | 0.014 | -0.109 | 0.136 | 0.8 |
| Total LEDD post-LCIG | 0.005 | -0.008 | 0.018 | 0.4 |
| D-Ag LEDD post-LCIG | -0.02 | -0.078 | 0.037 | 0.5 |
| UPDRS II | **0.536** | **0.038** | **1.034** | **0.035*** |
| UPDRS III | **0.516** | **0.216** | **0.817** | **0.001*** |
| UPDRS IV | 0.207 | -0.739 | 1.153 | 0.6 |
| HY | 3.755 | -0.899 | 8.409 | 0.1 |
| Time in ON post-LCIG | 0.325 | -2.263 | 2.913 | 0.8 |
| CCI | 0.463 | -4.464 | 5.39 | 0.851 |
| RDRS | -0.074 | -1.067 | 0.919 | 0.8 |
| WOQ-19 | -0.175 | -1.22 | 0.871 | 0.7 |
| WOQ-19 Motor | 0.001 | -1.76 | 1.762 | 0.9 |
| WOQ-19 Non-Motor | -0.519 | -2.086 | 1.048 | 0.5 |
| NMSS | **0.176** | **0.083** | **0.269** | **<0.0001*** |
| FOG-Q | 0.287 | -0.46 | 1.035 | 0.4 |
| PDSS-2 | 0.367 | -0.065 | 0.799 | 0.09 |
| ESS | 0.488 | -0.412 | 1.387 | 0.2 |
| QUIP-RS | -0.002 | -0.564 | 0.56 | 0.9 |
| ICD score | 0.147 | -0.557 | 0.852 | 0.7 |
| MOCA | -0.478 | -1.147 | 0.19 | 0.2 |

CCI= Charlson Comorbidity Index; CI= confidence interval; D-Ag= dopamine agonists; FOG-Q = freezing of gait questionnaire; EDS = Epworth Sleepiness scale; H&Y = Hoehn-Yahr stage; ICD = impulse control disorder; LCIG= Levodopa Carbidopa intestinal gel infusion; LEDD = levodopa equivalent daily dose; MOCA = Montreal Cognitive Assessment; NMSS = Non-Motor Symptoms Scale; PDSS-2 = Parkinson’s Disease Sleep scale-II; QUIP-RS= Questionnaire for Impulsive-Compulsive Disorders in Parkinson’s Disease–Rating Scale; RDRS = Rush Dyskinesia rating scale; UPDRS = Unified Parkinson’s Disease Rating Scale; WOQ-19 = Wearing Off Questionnaire 19 items. * Significant values are bolded.

**Supplementary table 2: Univariable regression with QUIP-RS after Levodopa-Carbidopa intestinal gel (LCIG) infusion as dependent variable**

|  | B | 95.0% CI for B | | P-Value |
| --- | --- | --- | --- | --- |
|  |  | **Lower Bound** | **Upper Bound** |  |
| Group (LE-PD vs Control-PD) | **-6.76** | **-11.256** | **-2.264** | **0.004*** |
| Age at onset | **-0.255** | **-0.501** | **-0.009** | **0.042*** |
| Age | **-0.358** | **-0.676** | **-0.041** | **0.028*** |
| Disease duration | 0.136 | -0.218 | 0.491 | 0.443 |
| LCIG duration | 0.03 | -0.033 | 0.093 | 0.342 |
| LEDD post LCIG | 0.003 | -0.004 | 0.009 | 0.431 |
| D-Ag LEDD post LCIG | 0.022 | -0.008 | 0.051 | 0.144 |
| UPDRS II | -0.039 | -0.308 | 0.23 | 0.771 |
| UPDRS III | -0.109 | -0.283 | 0.065 | 0.215 |
| UPDRS IV | 0.405 | -0.07 | 0.879 | 0.093 |
| HY | **-2.643** | **-4.988** | **-0.299** | **0.028*** |
| ON time post LCIG | -0.704 | -2.025 | 0.617 | 0.289 |
| NMSS | -0.041 | -0.094 | 0.012 | 0.129 |
| MOCA | 0.193 | -0.155 | 0.541 | 0.269 |

CI = confidence inerval; D-Ag = dopamine agonists; HY = Hoehn-Yahr stage; LE-PD = late elderly Parkinson’s disease; LEDD = levodopa equivalent daily dose; MOCA = Montreal Cognitive Assessment; NMSS = Non-Motor Symptoms Scale; PD = Parkinson’s disease; QUIP-RS = Questionnaire for Impulsive-Compulsive Disorders in Parkinson’s Disease–Rating Scale; RDRS = Rush Dyskinesia rating scale; UPDRS = Unified Parkinson’s Disease Rating Scale; WOQ-19 = Wearing Off Questionnaire 19 items. * Significant values are bolded.
